# Simultaneous bilateral total hip arthroplasty—a survey of Irish orthopaedic surgeons’ practice

**DOI:** 10.1007/s11845-024-03726-1

**Published:** 2024-06-05

**Authors:** Tom R. Doyle, Martin S. Davey, James P. Toale, Conor O’Driscoll, Colin G. Murphy

**Affiliations:** 1 Department of Orthopaedics, Galway University Hospital, Galway, Ireland; 2 Royal College of Surgeons in Ireland, Dublin, Ireland

**Keywords:** Simultaneous bilateral hip arthroplasty, Total hip arthroplasty, Total hip replacement

## Abstract

**Background:**

Bilateral total hip arthroplasty may be performed simultaneously (SIMTHA) or in two staged operations.

**Aim:**

To assess attitudes towards and utilization of SIMTHA in Irish orthopaedic practice, and to assess patient and surgeon factors which are associated with the management of bilateral hip arthritis.

**Methods:**

A 16-question electronic survey (Google Forms) was distributed via email to consultant Irish orthopaedic surgeons who perform total hip arthroplasty, followed by a reminder 1 month later. A *p* value < 0.05 was considered significant.

**Results:**

There were 53 responses from arthroplasty surgeons, with 28% reporting they never perform SIMTHA, 26% have performed ≤ 5 SIMTHA, and 46% do ≥ 1 SIMTHA per year. Amongst the 15 surgeons who do not do SIMTHA, 60% reported a preference for staged arthroplasty, 20% felt it was not feasible in their institution, and a third reported a lack of experience with SIMTHA. There was a significant association between not performing SIMTHA and years of consultant experience (*p* = 0.002). There were no institutional guidelines on eligibility criteria for SIMTHA. The most common time interval for staged bilateral arthroplasty was 6–12 weeks (60%). Overall, 56% of surgeons felt SIMTHA is underutilised in the Irish healthcare system; this was associated with greater SIMTHA volume (*p* = 0.023).

**Conclusion:**

Half of the Irish arthroplasty surgeons report SIMTHA is a regular aspect of their practice. Performing SIMTHA is associated with greater arthroplasty volume, more recent consultant appointments, and a perception that the operation is underutilised.

**Supplementary Information:**

The online version contains supplementary material available at 10.1007/s11845-024-03726-1.

## Introduction

Total hip arthroplasty (THA) has been hailed as the operation of the century and is used to address the significant morbidity of arthritis of the hip [1, 2]. The incidence of THA surgery is rising globally, with this trend projected to continue in the coming decades secondary to the ever-growing global population of older adults and people living with obesity [3, 4]. THA is the treatment of choice in cases of arthritis which have failed conservative measures, with the primary aim to alleviate pain, improve functional outcomes, and restore mobility [5]. However, bilateral arthritis is not uncommon, and patients with symptomatic bilateral degeneration may elect to have both hips treated under a single anaesthetic simultaneously (SIMTHA), or across two staged surgeries (STATHA). For patients with bilateral hip osteoarthritis who undergo a unilateral THA, approximately half will require a contralateral THA by 10 years [6–8], with between 20 and 25% of all elective THA recorded in European registries performed on the second contralateral hip [9].

SIMTHA was first described in 1971 by Jaffe and Charnley [10]; since then, there has been much debate with conflicting evidence regarding the outcomes from SIMTHA [11]. Studies and large systematic reviews have reported benefits of SIMTHA including significantly shorter length of hospital stay [12–14], reduced time under anaesthesia [15, 16], and reduced financial cost [17, 18]. Furthermore, the complication rate following SIMTHA has been reported as equal to that of STATHA [19, 20], with similar findings reported with respect to deep-vein thrombosis (DVT), pulmonary embolism (PE) [21], infection, and overall mortality [22, 23]. However, these findings have not been universal, and reporting from large registry studies has been important in identifying varied and conflicting reporting on the risk of transfusion, venothromboembolism (VTE), and mortality [8, 24–27].

There have been numerous systematic reviews comparing SIMTHA vs STATHA with major discrepancies observed in their conclusions, which likely stems from the conflicting evidence of the included clinical studies [28–32]. While to the best knowledge of the authors of this study, there are no guidelines published by any of the major orthopaedic internal societies to guide clinical practice with regard to SIMTHA. In the absence of clear evidence, the ideal treatment of bilateral hip arthritis remains unclear. Therefore, the purpose of this study was to assess attitudes towards and utilization of SIMTHA in Irish orthopaedic practice, and to assess patient and surgeon factors which are associated with the management of symptomatic end-stage bilateral hip arthritis.

## Methods

### Survey

A 16-question electronic survey was created using Google Forms (Appendix). The survey was distributed to consultant orthopaedic surgeons who as part of their routine elective practice perform total hip arthroplasty (THA) in the Republic of Ireland using the Irish Institute of Trauma and Orthopaedic Surgery emailing list. One month later, a follow-up email was sent as a reminder to increase the response rate. Surgeon experience and operating volume were assessed by asking the number of years of practice as a consultant and the number of THAs they perform per annum. The surgeons were asked how often they perform SIMTHA, with the following options: never, 1–5 times ever, 1–5 per year, > 5 per year, or regularly (weekly/monthly). These figures were selected following evidence from a registry study, which found improved outcomes in orthopaedic units who performed > 5 SIMTHA per year [33]. All participants provided digital informed consent prior to enrolment and all responses were anonymised at source. This study was deemed not to require ethical approval by our local ethics review board.

### Analysis

Anonymised survey data were analysed descriptively, and this is presented as a total number and percentage. Free text responses were assessed by two authors (TRD and JPT) using qualitative analysis following the grounded-theory approach [34]. Responses with a common theme were grouped and given an appropriate common code. The total number and percentage of theme-grouped answers are also presented. Statistics were performed using free open-source Jamovi statistical software (Jamovi version 2.3.28, available at www.jamovi.org). Categorical variables were analysed using a Fisher exact test or chi-square test. For continuous variables, a paired *t* test was performed to compare normally distributed variables, and the nonparametric Mann-Whitney *U* test or Wilcoxon signed-rank test was used for continuous variables. A *p* value < 0.05 was considered significant.

## Results

A total of 53 valid responses were received from Irish consultant orthopaedic arthroplasty surgeons. There was a mean of 13.8 ± 8.2 years of consultant experience. The mean THA operating volume was 142 ± 87 cases per annum. The most commonly reported surgical approach to THA was the posterior approach 57% (30), followed by the anterolateral approach (30%), with 7% reporting using a direct anterior approach and 6% using a direct lateral approach.

Overall, 15 consultants reported never performing SIMTHA (28.3%), while 14 surgeons have performed 1–5 SIMTHA during their career (26.4%). The remainder performs SIMTHA on a routine basis with 30.2% performing 1–5 per year, 9% performing > 5 a year, and 6% performing > 1 SIMTHA per month. Thus, 15% may be considered a high-volume SIMTHA surgeon [33]. Those with < 10 years of consultant experience were significantly more likely to perform SIMTHA compared to those with > 10 years of experience (88.9% vs 62.9%, *p* = 0.046). Eighty percent of those who perform > 100 THA also perform SIMTHA compared to 55.6% of those who perform < 100 elective THA per year, although this did not reach statistical significance (*p* = 0.765).

### Attitudes towards simultaneous bilateral total hip arthroplasty

Of the 15 surgeons who never performed SIMTHA, the most commonly reported reason was “surgical preference” in 60% of cases, while 33.3% felt they did have sufficient experience with the simultaneous procedure; 26.7% reported a lack of desire from their patients for the procedure, while 20% felt it was not feasible in their institution, as shown in Table 1.

**Table 1:** Surgeon reported reasons for not offering simultaneous bilateral total hip arthroplasty

**Response**	**%**
Surgical preference for staged arthroplasty	60.0
Lack of experience with simultaneous bilateral arthroplasty	33.3
Lack of patient desire	26.7
Not feasible in the surgeon’s institution	20

**Fig. 1:**
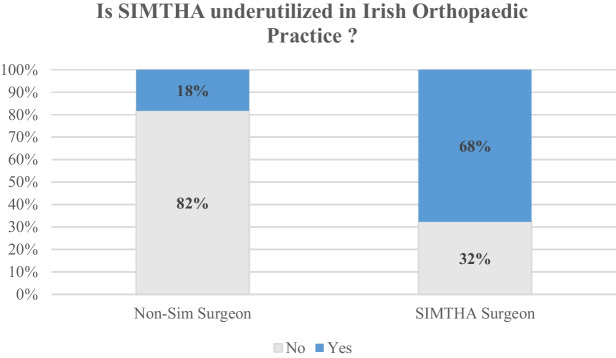
Attitudes towards utilisation of simultaneous bilateral total hip arthroplasty in Irish Orthopaedic practice

In total, there were 42 responses regarding the perceived potential benefits of SIMTHA. All surgeons agreed that a “single rehabilitation period” was of benefit. Other perceived benefits included a single exposure to anaesthesia (64.3%), the potential for a reduced length of stay compared to two admissions for staged bilateral arthroplasties (66.7%), and potential cost saving (31.0%) (Table 2 and Fig. 1).

**Table 2:** Surgeon reported benefits and negatives associated with simultaneous bilateral total hip arthroplasty

**Report benefits**	**%**	**Reported negatives**	**%**
Single rehabilitation period	100	Difficult postoperative rehabilitation	62
Single exposure to anaesthesia	64	Increased risk of minor complications	39
Reduced overall length of stay	67	Increased risk of major complications	36
Reduced overall cost	31	Increased risk of blood transfusion	8

There were 39 responses regarding the perceived potential negatives associated with SIMTHA. Overall, 61.5% reported that a perceived difficult postoperative rehabilitation period was negative. Other perceived negatives included the potential for a greater risk of minor complications (38.5%), risk of major complications (35.9%), risk of blood transfusion (7.7%), and risk of longer inpatient stay (5.1%), and 2.6% reported there were no associated negatives.

There were 48 responses (90.6%) regarding the question of whether SIMTHA is underutilised in Ireland. Overall, 56.3% of arthroplasty surgeons reported that they felt that SIMTHA is currently underutilised in Ireland. This was a polarising issue with 67.6% of surgeons who perform SIMTHA rating it as underutilised compared to just 18.2% of those who never perform the procedure (*p* = 0.004).

### Bilateral hip arthroplasty surgical practice

The 38 surgeons who perform SIMTHA reported the following as their personal relative contraindications: an elevated cardiovascular risk profile (59.0%), American Society of Anaesthesiologists grade (ASA) of ≥ 3 (53.8%), insulin-dependent diabetes (33.3%), age > 75 years (30.7%), patient not willing to accept increased risk of complications (7.9%), and technically difficult arthroplasty (2.6%). There were no institutions with formal inclusion/exclusion criteria for SIMTHA.

Seventy-nine percent of surgeons reported that they mandated review at a preassessment anaesthetic clinic (PAC) prior to SIMTHA. A further 7.9% reported that they had a lower threshold to send a patient to PAC than for a unilateral THA, while 13.1% reported no difference in PAC attendance compared to unilateral THA.

Overall, 44.7% of surgeons reported that they attempted to correct anaemia prior to SIMTHA. In total, 5.3% reported that they use cell saver technology during SIMTHA, while 52.6% reported that they do not take specific patient optimization measures prior to SIMTHA.

Eighty-four percent of arthroplasty surgeons report no difference in their postoperative VTE prophylaxis compared to unilateral arthroplasty, while 10.5% reported using more intensive prophylaxis for SIMTHA cases, and a further 5.3% reported using a prolonged period of prophylaxis.

The preferred interval for performing bilateral total hip arthroplasty staged across two operations was 6–12 weeks in 65.8% of surgeons, 12–24 weeks was preferred by 31.6%, and < 6 weeks was preferred by 2.1%.

## Discussion

SIMTHA has held appeal since first described by Charnley, for the potential to alleviate bilateral arthritis in a single visit to the operating room. However, the evidence base to guide surgical decision-making remains limited, and this is reflected in the heterogenous reports in the literature [28–30, 32]. In this representative sample of Irish arthroplasty surgeons, there was equipoise with 55% either never performing SIMTHA or have done < 5 in their career and 45% who do the procedure at least annually. Just over half the respondents feel the procedure is underutilised in Ireland, although two-thirds of surgeons who perform SIMTHA feel it is underused. Just 15% of respondents may be considered high-volume SIMTHA surgeons, which indicates there remains a relatively cautious uptake of the procedure. Of the surgeons who do not offer SIMTHA, a third reported this was due to a lack of experience with the operation; while higher arthroplasty volume and more recent appointments to consultant practice were associated with performing SIMTHA; this suggests that further uptake of the procedure is likely over the coming years.

Systematic reviews have suggested that SIMTHA is associated with significantly lower rates of DVT compared to STATHA [13, 28, 30, 32], while there is conflicting evidence across the literature with regard to the risk of PE, with reports of significantly higher and lower risk as well as reports of no difference [28, 30, 32]. In their 2018 systematic review, Muskus et al. declined to compare the two approaches as most studies were underpowered to detect a difference in VTE outcomes, as well as citing heterogenous methods of prophylaxis, outcome measurement, and patient risk profiles [29]. The two randomised control trial RCTs which compare SIMTHA vs STATHA found no difference regarding the risk of VTE, although they were underpowered to answer such questions [35, 36]. Recent large registry studies suggest there is an elevated risk of VTE after SIMTHA, although ultimately patient selection likely plays a large role via the VTE risk profile and level of postoperative mobility [11, 33]. This study has shown that the vast majority of Irish arthroplasty surgeons (84%) reported no difference in their respective VTE prophylaxis regimens compared to unilateral arthroplasty, while the remainder feel there is a substantial elevated risk with 11% prescribing more intensive prophylaxis and 5% prescribing for a prolonged duration.

Ideal patient selection for SIMTHA is a matter of debate and is likely the key determinant of positive outcomes after SIMTHA [21]. Age may be used as a cut-off with 70 and 75 years having been suggested [37, 38], others advocate for taking a broader view of patients’ health [31], while those with an ASA score of 3 may be at a higher risk of 90-day mortality [38]. This was largely reflected in the surgeons’ responses with over half using the ASA cut-off and a third using the age cut-off of 75. Pre-assessment is mandated by 80% of Irish surgeons and is an important time to optimise patients prior to major surgery. Almost half of the respondents reported ensuring pre-operative anaemia is corrected, although the true figure is likely higher given some surgeons likely defer this to a pre-assessment clinic. This is an encouraging practice to reduce the potentially higher risk of transfusions after SIMTHA [25, 35].

There was a clear lack of accepted definitions as to the ideal timeline of a STATHA. It has been reported that the second arthroplasty of a staged bilateral procedure should be avoided within 6 weeks of the primary operation due to the increased risk of myocardial infarction [39], while there is also evidence of those having staged operations during the same hospitalizations and within 6 weeks having inferior outcomes than SIMTHA [26, 38, 40]. Garland et al. compared in their registry data study STATHA with intervals of < 6 months, 6–12 months, and > 12 months to SIMTHA. They found no difference in mortality with a range of 0.1% between the groups at 90 days but with significantly lower mortality at 10 years in the SIMTHA cohort, highlighting the issues with the retrospective study in this area as SIMTHA patients are generally healthier than those undergoing STATHA [38]. The Poultsides et al. registry data study found a significantly higher rate of major complication when staged 6–12 months, and no difference between the 0–3- and 3–6-month groups [25] while the Partridge et al. registry data study compared staging intervals of < 3 months with 3–6 months and did not find a significant difference between morality or complications [33]. The most commonly reported staging interval in this survey was 6–12 weeks preferred by two-thirds of surgeons, while a third preferred an interval of 12–24 weeks, with just one surgeon aiming for < 6 weeks. The ideal staging interval is likely to be patient-specific, but currently, literature suggests that ideally the STATHA should be completed within 1 year of the index procedure, but after at least 6 weeks and once the patient has recovered from the first procedure.

A number of barriers to the adoption of SIMTHA were identified in this survey, with 20% surgeons who do not perform the procedure reporting that do feel it is feasible in the institution they work in. While there was universal agreement that a single period of rehabilitation was a benefit of the procedure, 60% also felt that the difficult nature of that rehab period was a negative. While the literature is clear that SIMTHA overall results in a shorter length of stay compared to two staged admissions, this is at the expense of a longer upfront admission and increased rates of discharge to rehabilitation facilities [11, 41]. In an Irish context, this presents an interesting dilemma, as many hospitals may not have the bed capacity to facilitate the slower turnover of SIMTHA patients, even if the total length inpatient stay will be shorter overall, while access to inpatient rehabilitation after discharge from the hospital remains a challenge [42]. As we move into an era of valued-based arthroplasty, there will be further pressure to optimise the cost of THA, and SIMTHA may be one option to help with this [43].

### Limitations

Limitations of this study include those inherent to level 5 evidence as the results are based on expert opinion. Although the responses were anonymous, they may be effected by the bias of self-reporting. While every effort was made to identify and contact all surgeons, it is possible that some were not identified, and as such, an exact response rate is not offered, while confirmation bias cannot be outruled and those with a greater interest in the topic may have preferentially responded.

## Conclusion

In this representative sample of Irish arthroplasty surgeons, approximately half report SIMTHA is a regular aspect of their practice, while treatment protocols for managing bilateral hip arthritis are highly heterogenous. Performing SIMTHA is associated with greater arthroplasty volume, more recent consultant appointment, and a perception that the operation is underutilised.

## Supplementary Information

Below is the link to the electronic supplementary material.

Supplementary file1 (PDF 612 KB)
