# Correction to: Simultaneous bilateral total hip arthroplasty—a survey of Irish orthopaedic surgeons’ practice

**DOI:** 10.1007/s11845-024-03755-w

**Published:** 2024-07-11

**Authors:** Tom R. Doyle, Martin S. Davey, James P. Toale, Conor O’Driscoll, Colin G. Murphy

**Affiliations:** 1 Department of Orthopaedics, Galway University Hospital, Galway, Ireland; 2 Royal College of Surgeons in Ireland, Dublin, Ireland

**Correction to: Irish Journal of Medical Science (2024)**

10.1007/s11845-024-03726-1

The original article contains an error. The last author’s middle initial should be change to “G” instead of “S”. The correct name should be “Colin G. Murphy”.

The original article has been corrected.

