# Supplementary material for: Simultaneous bilateral total hip arthroplasty—a survey of Irish orthopaedic surgeons’ practice
Source: Ir J Med Sci. 2024 Jun 5;193(5):2349–54. doi: 10.1007/s11845-024-03726-1 (PMC11450025; doi:10.1007/s11845-024-03726-1)
Supplement: Supplementary file 1 — Supplementary file1 (PDF 612 KB) [file 11845_2024_3726_MOESM1_ESM.pdf]

Attitudes Towards Bilateral Total Hip Arthroplasty Performed Simultaneously Vs Staged

A Cross Sectional Survey of Irish consultants.

* Indicates required question

1. **Q1** - Do you perform elective hip arthroplasty in your orthopaedic practice ? *

   Mark only one oval.

   - Yes
   - No

2. **Q2** - How many years experience as a consultant orthopaedic surgeon do you have ?

   Please enter the exact number in the section labelled other

   Mark only one oval.

   - 0-4
   - 5-9
   - 10-14
   - 15-19
   - >20
   - Other: ______________

3. **Q3** - What is your preferred surgical approach to performing total hip arthroplasty ?

Mark only one oval.

- Direct Anterior
- Anterolateral
- Lateral
- Posterior
- Other: ____________________

4. **Q3** - How many total hip arthroplasties do you perform per annum ?

Please enter the exact number in the section labelled other

Mark only one oval.

- 0-50
- 51-100
- 101-150
- 151-200
- 201-250
- 251-300
- >300
- Other: ____________________

5. **Q5** - How often do you perform simultaneous bilateral total hip arthroplasty ? *

   Please enter the exact frequency in the section labelled other

   Mark only one oval.

   - Never
   - A handful of times ever (1-5 operations in total)
   - Rarely perform this operation (1-5 operations a year)
   - Regularly perform this operation (>5 times a year)
   - Core component of your practice (weekly or monthly)
   - Other: ____________________

6. **Q6** - If the answer to the previous questions was "**never"**

   Why do you not perform simultaneous total hip arthroplasty ?

   Tick all that apply

   Tick all that apply.

   - [ ] It's not feasible to perform in my institution
   - [ ] Surgical preference
   - [ ] Lack of experience performing this procedure
   - [ ] Lack of patient desire for simultaneous bilateral arthroplasty
   - [ ] Other: ____________________

7. **Q7** - When performing simultaneous total hip arthroplasty do all patients attend a pre assessment clinic (PAC) first to assess fitness for surgery ?

   Mark only one oval.

   - Yes
   - No, but I have a lower threshold to send patients to PACS than for a unilateral arthroplasty
   - No difference in PACS attendance compared to a unilateral arthroplasty

8. **Q8** - When performing simultaneous bilateral total hip arthroplasty do you use ? Tick all that apply

Tick all that apply.

- [ ] Prior autologous blood donation
- [ ] Cell saver technology
- [ ] Preoperative correction of anaemia
- [ ] None of the above

9. **Q9** - When planning for staged bilateral total hip arthroplasty, what time interval do you aim for before the second operation ?

Mark only one oval.

- ◯ <1 week
- ◯ <6 weeks
- ◯ 6-12 weeks
- ◯ 12-24 weeks
- ◯ Cannot say - second hip is dependent on waiting lists and bed availability

10. **Q10 -** Does the VTE prophylaxis you use differ for Simultaneous Bilateral Arthroplasty compared to staged or unilateral arthroplasty

Mark only one oval.

- ◯ No difference
- ◯ Longer duration of propyhlaxis
- ◯ More intensive prophylaxis
- ◯ Other: ______________________

11. **Q11** - In your experience, what are the benefits of simultaneous bilateral total hip arthroplasty ?
Tick all that apply

Tick all that apply.

- [ ] Single exposure to anesthesia
- [ ] Potential for reduced overall length of hospital stay compared to staged bilateral operations
- [ ] Single rehabilitation period
- [ ] Cost reduction compared to staged bilateral arthroplasty
- [ ] Other: ____________________

12. **Q12** - In your experience, what are the negatives of simultaneous bilateral total hip arthroplasty ?
Tick all that apply

Tick all that apply.

- [ ] Difficult rehabilitation
- [ ] High rates of minor complications
- [ ] High rates of major complications
- [ ] Other: ____________________

13. **Q13** - Would any of the following factors convince you not to perform a simultaneous total hip arthroplasty ?
Tick all that apply

Tick all that apply.

- [ ] Age >75 years
- [ ] Higher anesthetic risk ASA grade >2
- [ ] Cardiovascular co-morbidity
- [ ] Diabetes
- [ ] None of the above
- [ ] Other: ____________________

14. **Q14** - Do you feel that simultaneous bilateral total hip arthroplasty is **underutilized** in Irish health care ?

Mark only one oval.

- Yes
- No

15. **Q15** - Do you or your institution have formalized inclusion/exclusion criteria for patient selection for simultaneous total hip arthroplasty ?

Mark only one oval.

- Yes
- No

16. **Q16** - If you or your institution have formalized inclusion/exclusion criteria for simultaneous total hip arthroplasty, please enter them below ?

____

This content is neither created nor endorsed by Google.

Google Forms
